# Repurposing the antihistamine cyproheptadine for osteoarthritis: nothing to sneeze at

**DOI:** 10.1172/JCI197144

**Published:** 2025-11-03

**Authors:** Richard F. Loeser, Philip R. Coryell

**Affiliations:** 1 Thurston Arthritis Research Center and; 2 Division of Rheumatology, Allergy and Immunology, School of Medicine, University of North Carolina, Chapel Hill, North Carolina, USA.

## Abstract

Osteoarthritis (OA) is a highly prevalent and painful joint disease in desperate need of disease-modifying therapeutics. Decline in the activity of the Forkhead box O (FOXO) family of transcriptional regulators in articular chondrocytes may contribute to the development of OA. In a study in this issue of the JCI, Kurakazu et al. screened compounds for FOXO activators and discovered that the antihistamine cyproheptadine activated FOXO3 through inhibition of the histamine H1 receptor. Cyproheptadine modulated the activity of OA-relevant pathways and reduced the severity of joint damage and pain behavior in a mouse model of OA, thus showing potential for development as a disease-modifying OA drug.

## Osteoarthritis and the need for disease modifying therapies

There is a critical need for disease modifying drugs for osteoarthritis (OA), a highly prevalent, chronic, and painful joint disease. OA is a leading cause of disability in adults. Globally, over 500 million individuals are affected by OA, and its prevalence continues to rise with the aging of the population as well as the obesity epidemic, which represent two important OA risk factors (1, 2). Current management is symptom focused and, for many patients, inadequate. Structurally, OA results from degradation and loss of the articular cartilage and is accompanied by bony sclerosis and osteophyte formation, varying degrees of synovitis, and damage to soft tissues within the joint. These soft tissues include ligaments and, in the knee, the menisci. As current therapeutics are unable to slow or stop joint tissue destruction, there is a growing need for joint replacement surgery for patients in whom symptomatic treatments have failed.

The many challenges to the development of new therapeutics that both reduce pain and modify structural damage in OA include insufficient in vitro models, the conduct of preclinical studies in young animals (which are less translationally relevant to the typical age when humans develop OA), and the heterogeneity of OA patients (3). OA develops over many years, making it difficult to know when to intervene, and is usually not symptomatic until advanced stages of structural changes are present. In addition, a multitude of risk factors for OA, including joint injury and shape, sex, genetics, advanced age, and obesity likely result in multiple OA phenotypes and endotypes that may respond differently to specific interventions (4).

## Repurposing cyproheptadine in OA

Once considered simply a “wear and tear” condition, the past 10–20 years of research have resulted in substantial advances in understanding the mechanisms underlying OA structural damage as well as pain. The results presented in the featured study by Kurakazu et al. (5) represent another advance as well as an exciting new approach to therapy. Kurakazu and colleagues discovered that inhibition of signaling mediated by the histamine H1 receptor (HRH1) using the antihistamine cyproheptadine can prevent the development of structural damage and pain in a mouse OA model. The action of cyproheptadine was connected to a unique set of mechanisms that included activation of the transcriptional regulator Forkhead box O 3 (FOXO3) as well as FOXO3-independent effects on proinflammatory signaling. Cyproheptadine and desloratadine, which demonstrated similar effects in this work, are inverse agonists of HRH1; unlike antagonists that block ligand/receptor binding to prevent activation, inverse agonists reduce constitutive receptor activity that is present even in the absence of ligand binding. Given that these HRH1 inverse agonists are already on the market for the treatment of allergies and related conditions, the possibility of repurposing these relatively safe drugs for the treatment of OA brings new hope. However, more work will be needed to determine if HRH1 inhibition will translate into benefits for older adults with established OA, the group with the greatest need.

## Role of reduced FOXO activity in OA

FOXO transcription factors are key regulators of cellular stress, autophagy, and antioxidant defenses in many tissues. FOXO activity requires its translocation from the cytoplasm to the nucleus. FOXO1 and FOXO3, but not FOXO4, are abundantly expressed in superficial and mid-zone chondrocytes and are mostly localized to the nucleus (6). Aging has been associated with reduced FOXO1/3 expression in human and mouse cartilage, and OA has been associated with a marked reduction in FOXO1/3 nuclear localization (6). In cultured chondrocytes, FOXO1 nuclear translocation was inhibited by the cytokines IL-1β and TNF-α and increased by the growth factors TGF-β and PDGF (6). Triple deletion of *FoxO1/3/4* in chondrocytes strongly decreased expression of autophagy genes, antioxidant defense genes, and genes related to redox regulation and adaptation to energy stress (7). Furthermore, single *FoxO*-knockout mice, particularly *FoXO1* but also *FoXO3*, exhibited more severe spontaneous OA, reduced expression of *Prg4* (encoding the cartilage surface protein lubricin), and increased cartilage degradation in the mouse destabilized medial meniscus (DMM) model of OA (7). Conversely, overexpression of FOXO1 resulted in increased levels of autophagy genes, increased *Prg4* expression, and prevented IL-1β–induced inflammatory and catabolic mediators of OA. Collectively, the data indicate that FOXO is an essential regulator of joint health and homeostasis via its ability to regulate cellular stress and viability in articular cartilage, and that its activity is severely limited in OA.

Given the protective roles of autophagy and FOXO function in articular cartilage, several therapeutic strategies are under consideration to restore both of these factors in OA. Prior to Kurakazo et al.’s study, this research group’s compound library screen for FOXO activators discovered that histone deacetylase inhibitors (e.g., Panobinostat) inhibited production of OA mediators in vitro and reduced the severity of OA in the mouse DMM model (8). Inhibiting histone deacetylases may not be the best approach for treating OA, given the complex biology around histone function, so Kurakazo and colleagues extended the work and looked for alternative FOXO activators, leading to the discovery of cyproheptadine (5). Another group has taken a different approach, using intra-articular delivery of a lentivirus expressing FOXO3, which activated autophagy and reduced subchondral bone remodeling and cartilage degeneration induced by high-fat diet in mice (9). Together, these studies support targeting FOXO activity as a promising therapeutic strategy for OA.

## Histamine and histamine receptors in OA

Studies have shown that histamine is present in the synovial fluid that bathes the joint, and its origin has been attributed to histamine-producing mast cells observed in the synovium (10, 11). Indeed, mice lacking mast cells were protected from the development of OA in the DMM model (12). However, mast cells produce a number of mediators in addition to histamine, such as the serine protease tryptase, which could also contribute to OA. There is in vitro evidence that histamine can stimulate chondrocytes to produce matrix-degrading enzymes, including the metalloproteinases MMP-3 and MMP-13 (13). Moreover, histamine activation of chondrocyte H1 and H2 receptors was shown to potentially promote joint tissue degradation through signaling that resulted in activation of RANKL and NR4A, components of an essential pathway that regulates bone resorption and is closely linked with OA (14).

**Figure 1:**
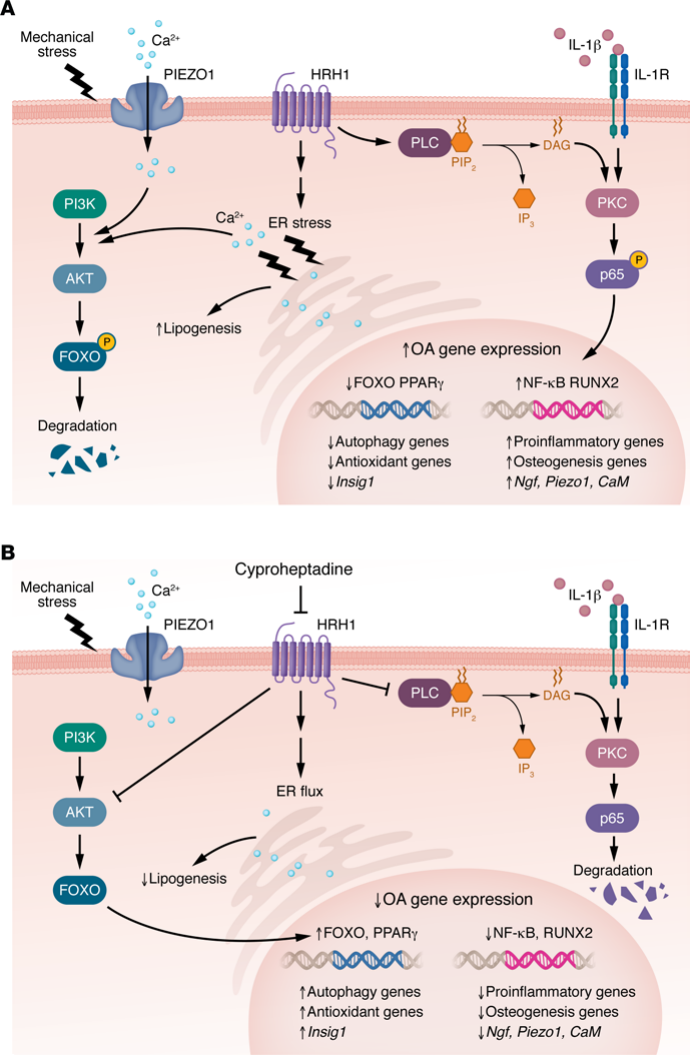
HRH1-mediated signaling in OA and the effects of cyproheptadine inhibition. (**A**) In OA, constitutive HRH1 signaling contributes to ER stress and increased release of calcium (Ca2^+^) to the cytosol, which serves to stimulate AKT activation. AKT activation leads to phosphorylation of FOXO, causing it to be retained in the cytosol where it is degraded. The resulting decreased levels of FOXO in the nucleus decrease expression of FOXO-regulated genes. HRH1 signaling also activates PLC, which leads to PKC activation and increased p65 phosphorylation, which promotes p65 translocation to the nucleus, increasing transcription of proinflammatory NF-κB–mediated genes. HRH1 also promotes IL-1 signaling as well as an increase in RUNX2-mediated transcription of osteogenesis genes that could promote bone formation seen in OA. Other genes upregulated by HRH1 include those coding for the pain mediator NGF, the calcium channel protein Piezo1, and the calcium binding protein calmodulin. Additional effects of HRH1 include downregulation of PPARγ activity and Insig1 expression, the latter of which results in increased lipogenesis. (**B**) Cyproheptadine, acting as an inverse agonist, inhibits HRH1 signaling, resulting in increased ER flux. Enhanced ER flux balances calcium levels in the cytosol and increases activity of FOXO3. It also inhibits proinflammatory signaling to reduce expression of OA mediators, an effect also mediated by the increase in FOXO3 and PPARγ.

The present study by Kurakazu et al. (5) identified a mechanism by which constitutive activity of HRH1 in chondrocytes, independent of the presence of histamine, resulted in activation of signaling that inhibited FOXO activity and upregulated expression of OA mediators (Figure 1A). They noted that HRH1 was the primary histamine receptor expressed by chondrocytes and that its levels were increased in human OA cartilage. Inhibition of chondrocyte HRH1 signaling by cyproheptadine altered activity of several different pathways, in part, due to alterations in calcium flux between the endoplasmic reticulum and the cytosol. The alteration in calcium signaling resulted in a reduction in phosphorylation of the signaling protein AKT, which normally promotes FOXO retention and degradation in the cytosol. Through this mechanism, cyproheptadine promoted FOXO3 nuclear translocation to increase expression of its target genes (Figure 1B), resulting in improved autophagy and an increase in the intracellular antioxidant status.

Many but not all of the effects of cyproheptadine were due to increased FOXO3 activity. Inhibition of NF-κB–mediated proinflammatory gene expression was independent of FOXO3 activation. Cyproheptadine also inhibited IL-1–driven proinflammatory signaling in chondrocytes and synovial fibroblasts, which is thought to play an important role in OA pathogenesis. This finding suggests a unique cross talk occurring between HRH1 and IL-1 signaling. Cyproheptadine also blocked IL-1–induced expression of nerve growth factor (NGF), an important pain mediator in OA, and increased expression of PPARγ target genes, which would also be predicted to benefit joint tissue homeostasis (15). Other FOXO3-independent effects included increased insulin induced gene 1 (*Insig1*) expression, which suppressed lipogenesis and cholesterol metabolism; inhibition of ossification gene expression in chondrocytes and mesenchymal stem cells, mediated by a decrease in RUNX2 activity; and reduced expression of PIEZO1, a mechanosensitive ion channel protein that mediates calcium influx and is also thought to contribute to pain and inflammation in OA (16).

As noted above, cyproheptadine and similar antihistamines have been in clinical use for many years. Epidemiologic studies examining chronic antihistamine use and OA risk have shown mixed outcomes. Antihistamine use was associated with reduced prevalence of knee OA in a cross-sectional study using data from the Osteoarthritis Initiative (OAI) (17), and a separate longitudinal OAI study noted a “weak signal” for reduced OA progression (18). In contrast, another OAI study failed to find evidence that antihistamines were protective of knee OA radiographic progression (19), nor was a benefit seen for prevalent OA, joint pain, or future incident OA in a large Swedish cohort (20). However, these studies included all antihistamines — antagonists as well as inverse agonists of histamine receptors. Inhibition of HRH1 constitutive activity by an inverse agonist such as cyproheptadine may be more effective in OA than receptor antagonists that block ligand binding without affecting receptor activity. It is not clear if antihistamines with mechanisms that differ from cyproheptadine or desloratadine would have the same effects as those reported by Kurakazu et al. (5).

## Conclusions and future directions

The study by Kurakazu et al. (5) revealed mechanisms by which structural damage and pain can develop in OA that unexpectedly involved constitutive signaling mediated by a histamine receptor HRH1, which is expressed by chondrocytes and synovial fibroblasts and is increased in OA cartilage. An inverse HRH1 agonist, cyproheptadine, increased FOXO3 activity but also inhibited proinflammatory signaling independent of FOXO3 and modulated the activity of a host of OA mediators, resulting in the prevention of OA development in the DMM mouse model.

Further studies are needed to translate this therapy to benefit humans, starting with studies to determine if treatment with cyproheptadine can slow or stop the progression of established OA. Since the studies by Kurakazu et al (5) were performed in young 15-week-old male mice, experiments are needed using animals with established OA at an age more similar to when humans develop symptoms (e.g., 6–12 months of age in mice). Moreover, future studies should include female mice, as human OA is more prevalent in women. The mixed results of antihistamine use and OA risk in observational studies indicate that randomized, placebo-controlled trials of specific antihistamines are needed. Because there are multiple OA phenotypes and endotypes, studies will also be needed to determine if targeting HRH1 will benefit OA subtypes other than injury-induced posttraumatic OA, which was modeled in the DMM experiments. Given the safety profile of antihistamines such as cyproheptadine, repurposing these drugs for systemic treatment of OA could be of great benefit to not only individuals with knee OA but potentially other joints as well, resulting in a tremendous advance in the management of the many individuals suffering from OA.

## Funding support

This work is the result of NIH funding, in whole or in part, and is subject to the NIH Public Access Policy. Through acceptance of this federal funding, the NIH has been given a right to make the work publicly available in PubMed Central.

- National Institutes of Health (R01AR079538 and R01AG044034 to RFL).
- Rheumatology Research Foundation.
- Department of Defense.

## Version 1 11/03/2025

Electronic publication
